# Voice Assessment in Patients With Obstructive Sleep Apnea Syndrome After Transoral Robotic Surgery

**DOI:** 10.3389/fsurg.2021.647792

**Published:** 2021-03-17

**Authors:** Hsin-Hsin Huang, Chien-Han Tsao, James Cheng-Chung Wei

**Affiliations:** ^1^Institute of Medicine, Chung Shan Medical University, Taichung, Taiwan; ^2^Department of Otolaryngology, Chung Shan Medical University Hospital, Taichung, Taiwan; ^3^School of Medicine, Chung Shan Medical University, Taichung, Taiwan; ^4^Department of Allergy, Immunology and Rheumatology, Chung Shan Medical University Hospital, Taichung, Taiwan

**Keywords:** transoral robotic surgery, multi-dimensional voice program, real time pitch program, voice handicap index-10, pitch, semitone

## Abstract

**Objectives:** Removal of part of the tongue base, in combination with uvulopharyngopalatoplasty via transoral robotic surgery (TORS), for treating obstructive sleep apnea syndrome (OSAS) results in enlargement of the oropharynx and hypopharynx and change in the size of the resonance chamber. These procedures may also alter the laryngeal-hyoid bone complex, which is linked to vocal fold tension. Thus, there is the potential for change in phonation and pitch after surgery.

**Study Design:** Prospective, nonrandomized, institutional board-approved study.

**Methods:** From January to August 2018, 15 patients with OSAS receiving TORS underwent voice and pitch sampling. The multi-dimensional voice program (MDVP) was applied to the evaluation of preoperative sound parameters. Highest pitch and lowest pitch were obtained with real-time pitch software, with pitch synchronized to electronic organ or tuner. Subjects also completed the Voice Handicap Index-10 scale (VHI-10), to assess their subjective perception and to detect factors affecting the VHI-10 score. The relevant parameters were analyzed again 3 months after the operation.

**Results:** There was an increase in VHI-10 score 3 months after operation that did not reach statistical significance. There were also no significant differences in sound parameters. Increases in highest pitch (353.18 Hz shift to 387.99 Hz), highest semitone (${\text{F}}_{5}^{\#}$ shift to ${\text{F}}_{5}^{\#}$), lowest pitch (117.45 Hz shift to 131.42 Hz), and lowest semitone (C_3_ shift to C_3_) did not reach statistical significance. The increase in the lowest semitone was significantly related to change in VHI-10 score (*r* = −0.808, *P* = 0.028).

**Conclusion:** Patients with OSA undergoing TORS showed a negative correlation coefficient over 0.8 with change in VHI-10 score. That is, increase in the lowest semitone after operation correlated with increase in VHI-10 score which may cause perceive changes in subjective pronunciation.

## Introduction

Patients with obstructive sleep apnea syndrome (OSAS) experience repeated suffocation and poor ventilation due to the collapse of upper respiratory tract soft tissues during sleep. Excision and remodeling of soft and hypertrophic tissues in the oropharynx and hypopharynx serve to expand the upper respiratory tract. The effectiveness of transoral robotic surgery (TORS) for OSAS has been confirmed by imaging and polysomnography (PSG) (1, 2). However, whether surgical removal and reset of benign tissues, which assist in swallowing, taste perception, and sound resonance, change the quality of life of patients or affect patients professionally is an important question. In the 20 years from 1993 to 2003, there was increasing prevalence of sleep apnea (3). In addition to increasing numbers of consultations, clinicians should be alert to the rise in patients' rights awareness.

Under the concept of multi-level surgery, TORS for OSAS provides therapeutic benefits for sleep apnea patients (1, 2) and changes the size of the resonance chamber (1). Does the partial removal of the genioglossus muscle affect the position of the hyoid bone, which in turn affects the tension of the vocal cords (4, 5) and pronunciation? The aim of this study is to attempt to answer this question.

## Materials and Methods

### Study Design

Fifteen patients diagnosed with OSA proven by polysomnography (PSG) and normal hearing proven by pure tone audiogram (PTA) were enrolled in this study from January to August 2018. They each signed an informed consent form and underwent sound evaluations pre- and post-surgery. Multi-dimensional voice program (MDVP) is a voice parameter analytical tool (6) for speech sampling, phonation, and standard pitch. In addition to traditional sound parameters, changes in pitch were included in the analyses. An electronic organ, a tuner, and real-time pitch software were used to measure highest pitch and lowest pitch. Patients also completed the Voice Handicap Index-10 scale (VHI-10) (7), which assesses patients' subjective perception of voice changes. Sleep parameters, sound parameters, and VHI-10 score were checked again 3 months after the operation.

### Measuring Tools and Methods

MDVP (Kay CSL4500 model, Lincoln Park, NJ, USA) and real-time pitch software Sound Blaster Live Value by Creative Technology Limited were used for sound analysis. Additional equipment included SHURE SM48 microphone and Core Duo dual core (2.93 GHz) processor. Electronic organ and tuner were approved by the National Bureau of Standards.

The microphone was placed about 15 cm from the oral cavity while each patient read a sentence in Chinese aloud for speech sampling. The collected parameters included fundamental frequency (Fo), mean fundamental frequency (MFo), pitch period perturbation quotient (PPQ), frequency disturbance ratio (Jitter, %), amplitude disturbance ratio (Shimmer,%), amplitude perturbation quotient (APQ), noise-to-harmonic ratio (NHR), voice disturbance index (Voice), turbulence index (VTI), and soft phonation index (SPI) (8). Before measuring pitch, all subjects received vocal training from a professional language therapist. The length of the training course depended on patient sensitivity to scales and singing experience, but all subjects received at least one session.

Tools for measuring pitch included real-time pitch software, electronic organ, and tuner. Individuals who completed the vocal training course listened to semitones emitted from electronic organ. Each patient was asked to sing an octave while pronouncing the sound “Ah” from center C. With the help of a speech therapist, each patient sang the notes played, such as center D, center E, center F, center G, center A, center B, and treble C. The frequencies were 262, 294, 330, 349, 392, 440, 494, and 524 Hz. Each note was sung 3 times. Once the patient was familiar with the center octave, he or she was asked to extend from central C to treble octave; treble C, treble D, treble E, and treble F, singing down the scale in the same pattern, while holding each semitone for 3 s (8). Under MDVP monitoring, Jitter of <0.6% and Shimmer of <2.4% were used to assess best pitch (9). Otherwise, there was dropping or elevation of one semitone to determine the highest pitch and lowest pitch.

The parameters included the lowest pitch and the highest pitch synchronized with electronic tuner. The electronic tuner divided the gap between each semitone into 100 equal parts. For example; if the pitch measured by tuner was G5 minus 20, the frequency was 784.006-20x [(784.006-740.074)/100] = 775.222 Hz. The frequency calculated by subtracting 20 from A3 was 229.261-20x [(229.261-220.071)/100] = 227.423 Hz. When a patient's pitch was stable, this fit the criteria of MDVP (Jitter <0.6%, Shimmer <2.4%). Results from use of electronic tuner were regarded as the first record. The second test was conducted using real-time pitch software. There was little difference between the frequency obtained with electronic tuner and that obtained with real-time pitch software. The frequencies acquired with these two methods were averaged and used as pitch data. Three patients with normal hearing were unable to produce equal-frequency pitches by listening to the played semitones. In these patients, pitch range was obtained using timely pitch software.

**Figure 1:**
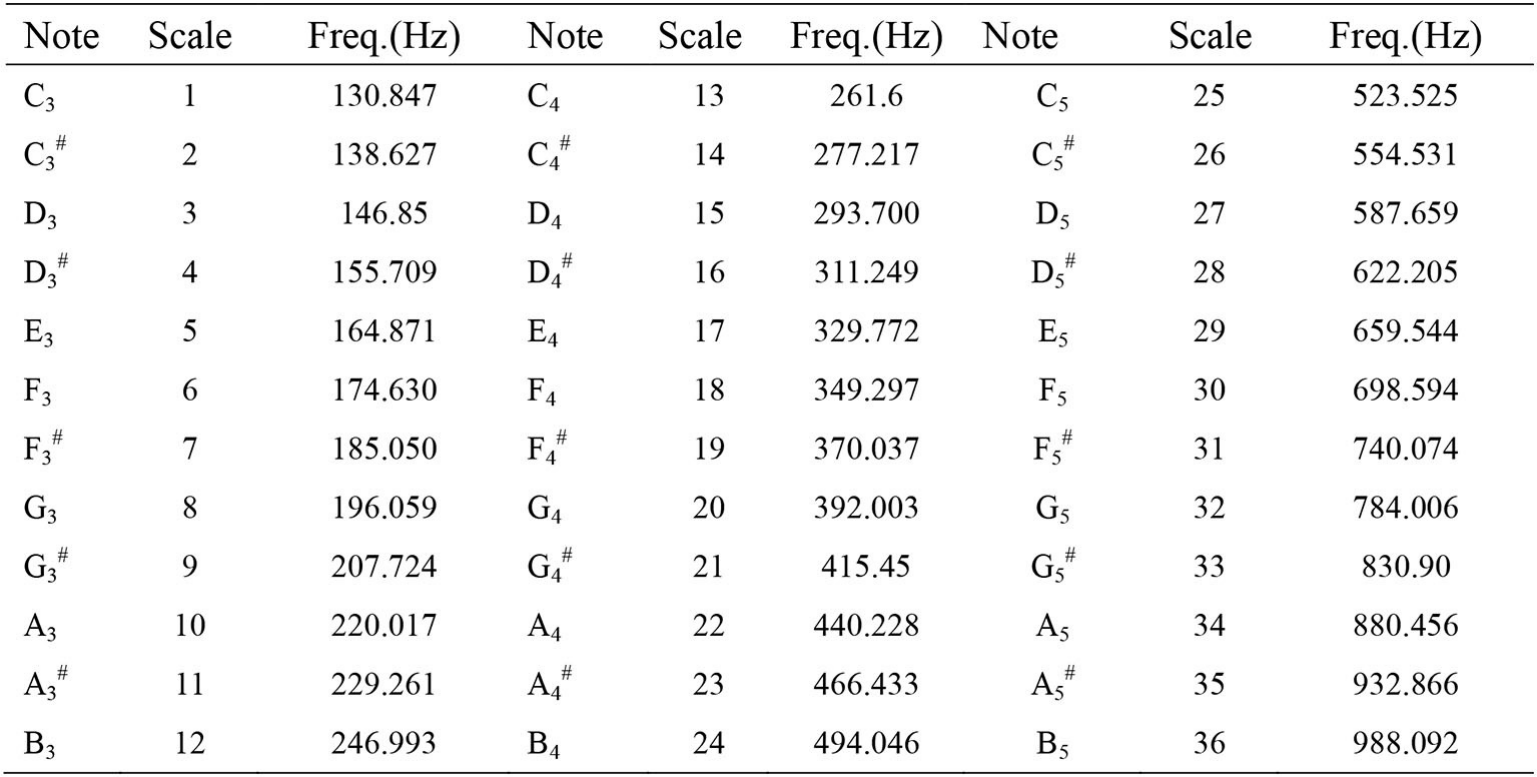
Musical note and scale to frequency chart. According to an electronic keyboard played from left to right in a standard soundproof room with multi-dimensional voice analysis software to measure and obtain the corresponding frequency, retaining all decimal points.

Subsequently, the recorded frequencies were converted into the lowest semitone (lowest note), the highest semitone (highest note), and the semitone range (note range). Semitones were recorded using the discarding method and lower than standard semitones were downgraded. If the tuner-detected pitch was E5 minus 20, D5 # and 28 were recorded (Figure 1). Figure 1 shows the laboratory measurements of the relative frequencies of keyboard pitch using multi-dimensional voice analysis software in a standard soundproof room, with a total of 36 semitones ranging from C3 to B5. Position C3 was denoted as 1 and position B5 was denoted as 36. Degree of note decline before and after surgery was recorded. Subjective evaluation of vocal changes was carried out using VHI-10 (9).

### Statistical Analysis

The software package SPSS 22nd edition (SPSS, USA) was used for statistical analysis. The changes in sound parameters were examined by Wilcoxon signed-rank test. Spearman rank correlation was used to correlate the changes in sound parameters, pitch, and posterior voice disorder index. Two-tailed tests were used with *p* < 0.05 considered statistically significant.

### Surgical Procedure

**Figure 2:**
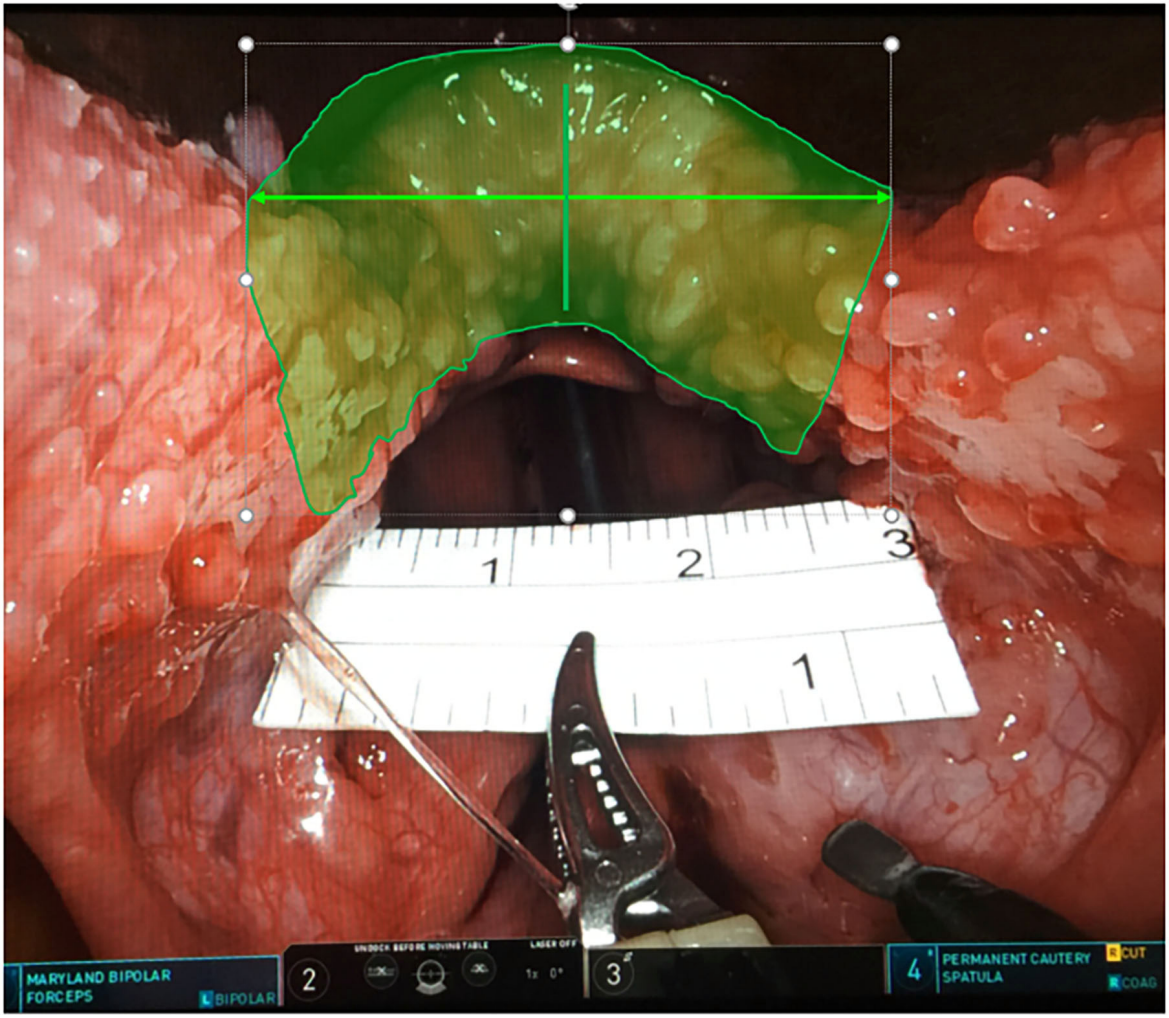
Surgical range on base of tongue.

All cases underwent TORS at the base of the tongue combined with uvulopalatopharyngoplasty (UPPP) and laterolpharyngoplasty. Endotracheal intubation from the nose was carried out with two 3.0 silk threads through anterior central sides of the tongue body to draw out the tongue without winding around the tongue tip, to avoid the pressure of the mouth opener and silk thread. The Larynx Advanced Retractor System (LARS) was used to open the mouth. Generally, the long blade is changed to short blade and adjusted in the center of the tongue. Visually, the two sides of the tongue are symmetrical under the blade and the junction between the front pillar and the tongue is exposed. The robotic arm system used in this surgical procedure was “da Vinci Surgical System, Si” (Intuitive Surgical Company. Sunnyvale, California, USA), with unipolar cutting on the right and bipolar electric burning (Bipolar Maryland dissecting forceps) on the left. A ruler was used to measure the total width of the 3 cm tongue root excision. The left and right sides were 1.5 cm from the midline, the depth was close to 1 cm, and the central 1 cm was deepened by a maximum of 0.5 mm. Left partial tongue base resection was performed first, followed by right side tongue base resection, according to the method of D'Agostino. (10) (Figure 2). Re-docking in this surgical procedure is not necessary. After reduction of the left and right tongue bases, right side tonsillectomy and suspension pharyngoplasty were performed. While carrying out the procedures on the left side, the two instruments in the left and right hands were exchanged. This study was approved by the IRB of the Human Experiment Committee of Chung Shan Medical University (CS-17104).

## Results

Table 1 presents the demographic characteristics of the 15 subjects in this study. The average age was 46.48 ± 11.37 years. Males accounted for 80% (12/15) of the subjects. Physical fitness index was 86.6% (13/15) ≥27 kg/m^2^. Of the 15 sleep apnea patients, 11 (73.3%) were severe and 3 (20%) were moderate.

**Table 1:** Demographic data of OSA patients.

**Variable**	***n*(%)**
Age (years)	46.48 ± 11.37
Sex (F)	15 (2)
Smoking (%)	6 (40%)
Drinking (%)	1 (6.6%)
Obesity^a^ (%)	13 (86.6%)
Hypertension (%)	13 (86.6%)
Diabetis mellitus^b^ (%)	3 (20%)
Hyperlipidermia^c^ (%)	8 (53.3%)
Obstructive sleep apnea^d^	15
Mild	1(6.6%)
Moderate	3(20%)
Severe	11(30)

*Expressed as a numerical value (percentage) or average ± standard deviation*.

a *Obesity: body mass index BMI value = 27 kg / m^2^*.

b *All diabetic patients suffered from type 2 diabetes*.

c *Hyperlipidemia: total serum cholesterol level ≥200 mg / dl, triglyceride level (serum triglyceride level) ≥200 mg / d or taking drugs to control blood lipids*.

d *Obstructive sleep apnea: defined as sleep disordered breathing index (AHI, apnoea-hypopnoea index) ≥ 5*.

Table 2 shows the changes in sleep parameters before and after surgery in 15 cases. Apnea hyponia index (AHI) decreased from 51.54 times/h to 34.31 times/h, lowest blood oxygen concentration (Lowest SPO2) increased from 73.50 to 80.57%, and snoring index (Snoring index) decreased from 444.5 times/h to 366.51 times/h.

**Table 2:** Differences in sleep parameters before and after TORS.

**Parameter**	**Pre-op** **(*n* = 15)**	**Post-op** **(*n* = 15)**	***P-*value**
Age (years)	46.48 ± 11.37		
Sex (male %)	25(30)		
BMI (kg/m^2^)	27.52 ± 3.60	27.34 ± 2.926	0.519
Polysomnography (PSG)			
Apnea-hyponea index (AHI, events/h)	51.54± 25.29	34.31 ± 22.80	0.000
Total sleep time, (h)	8.86 ± 1.73	7.34 ± 2.03	0.911
Sleep efficiency, (%)	88.87 ± 8.49	90.28 ± 7.817	0.442
Sleep latency, (min)	114.54 ± 57.39	103.43 ± 43.92.	0.411
Sleep architecture, (% of total sleep time)			
Rapid eye movement (REM, %)	17.72 ± 7.00	18.80 ± 6.23	0.453
Stage 1 sleep, (%)	27.42 ± 17.03	21.66 ± 15.66	0.040
Stage 2 sleep, (%)	58.32 ± 12.85	53.11 ± 17.55	0.139
Stage 3 sleep, (%)	1.08 ± 2.46	1.27 ± 4.11	0.830
Stage 4 sleep, (%)	0.00 ± 0.00	0.00 ± 0.00	
Arousal index, (event/h)	33.36 ± 21.29	30.18 ± 20.47	0.426
Oxygen desaturation index (ODI, event/h)	44.22 ± 26.06	24.15 ± 22.04	0.000
Longest apnea time, (s)	48.70 ± 17.23	41.09 ± 25.22	0.142
Mean SaO2, (%)	91.74 ± 15.97	94.09 ± 2.13	0.43
Lowest SaO2, (%)	73.50 ± 11.42	80.57 ± 9.70	0.000
Snoring index, (events/h)	444.56 ± 101.61	366.51 ± 146.42	0.008

*BMI, Body mass index; PSG, Polysomnography; AHI, Apnea Hypopnea Index; REM: Rapid eye movement; ODI, Oxygen desaturation index*.

Table 3 presents the results of surgery according to the various definitions of success. Cure rate was 6.6%. Moreover, 36.6% of cases no longer required the use of CPAP. AHI value in 26.6% of cases was reduced by 50%. A total of 63.3% of cases achieved traditional definition of success.

**Table 3:** Surgical results for different AHI.

**Criteria**	**Definition**	**Proportion**	**Cumulative**
Cured	AHI <5 and ESS <10 and >50% reduction	2/15 (6.6%)	6.6%
No need to use CPAP	AHI <15 and ESS <10 and >50% reduction	5/15 (33.3%)	39.9%
Success	AHI <20 and ESS <10 and >50% reduction (reduction/elimination of excessive daytime somnolence and cardiovascular risk factors)	4/15 (26.6%)	66.5%
Failure	AHI >20 and any ESS value and <50% reduction (no real clinical improvement)	4/15 (26.6%)	26.6%

Table 4 presents the objective sound parameters. The differences in the objective sound parameters of these 15 patients before surgery and 3 months after surgery did not reach statistical significance. The highest pitch, pitch range, highest note, and note grade range were compared before and after surgery, with no significant changes. The subjective voice impairment index showed an increase from 7 ± 7.37 points before surgery to 8 ± 8.52 points 3 months after surgery. However, this difference was not statistically significant.

**Table 4:** Comparisons of acoustic parameters, common pitch, and VHI-10 scale, pre and post TORS for OSAS.

**Parameters**	**Pre-operation**	**Post -operation**	P -value^*^
**Multi-dimensional voice program,**			
**MDVP**			
Fo (Hz)	141.53 ± 28.73	135.56 ± 35.51	0.553
MFo (Hz)	139.01 ± 27.63	133.13 ± 34.20	0.540
PPQ (%)	2.00 ± 0.54	2.31 ± 0.62	0.389
Jitter (%)	3.18 ± 0.80	3.62 ± 0.98	0.381
Shimmer (%)	7.58 ± 3.22	10.83 ± 2.81	0.080
APQ (%)	8.49 ± 4.65	12.86 ± 3.71	0.145
NHR (%)	0.20 ± 0.55	0.26 ± 0.41	0.093
VTI	0.23 ± 0.33	0.16 ± 0.25	0.251
SPI	24.87 ± 4.78	24.39 ± 3.27	0.776
**Pitch and note**			
Lowest pitch (Hz)	117.45 ± 30.45	131.42 ± 48.44	0.334
Highest pitch (Hz)	353.18 ± 161.92	387.99 ± 143.59	0.167
Pitch range (Hz)	235.72 ± 156.70	256.57 ± 104.30	0.449
Lowest note	1.57 ± 1.51	2.57 ± 3.74	0.281
Highest note	15.7 ± 8.90	18.7 ± 5.74	0.156
Note grade range	14.14 ± 8.23	16.14 ± 3.76	0.386
VHI 10	7 ± 7.37	8 ± 8.52	0.742

* *Wilcoxon signed rank test. All parameter values in the table are expressed as mean ± standard deviation. Fo(Hz), Average fundamental frequency; MFo, Mean fundamental frequency; PPQ, Pitch period perturbation quotient; Jitter, Jitter percent; Shimmer, Shimmer percent; APQ, Amplitude Perturbation Quotient, NHR, Noise to Harmonic Ratio; VTI, Voice turbulence index; SPI, Soft phonation index; RAP, Relative Average Perturbation*.

From Table 5, the shifts in objective sound parameters caused changes in VHI-10 scores. Increase in the lowest semitone showed significant correlation with increase in VHI-10 score (r = −0.808, *P* = 0.028).

**Table 5:** Correlation of VHI-10 score with pitch and sound parameters.

	**VH-10 scores before and after surgery**	
**Parameters**	**Correlation coefficient**	*P* value^*^
Fo (Hz)	−0.424	0.344
MFo (Hz)	−0.461	0.297
PPQ (%)	−0.331	0.468
Jitter (%)	−0.517	0.235
Shimmer (%)	−0.311	0.498
APQ (%)	−0.276	0.549
NHR (%)	−0.034	0.943
VTI	0.115	0.806
SPI	−0.425	0.342
Lowest pitch (Hz)	−0.457	0.799
Highest pitch (Hz)	−0.297	0.518
Pitch range (Hz)	−0.020	0.996
Lowest note	−0.808^*^	0.028
Highest note	−0.040	0.932
Note grade range	0.285	0.536

* *Spearman's rho test*.

## Discussion

This is the first study on voice assessment after TORS for OSA. The area influenced includes the oropharynx, hypopharyngeal cavity, and even the larynx. In 2010, scholars from the Medical College of South Valley University in Egypt performed acoustic analyses on 29 cases undergoing UPPP and laser-assisted uvulopalatoplasty (LAUP). After UPPP, there was no meaningful change in Jitter, Shimmer, or NHR (11). Oropharyngeal surgery does not affect the vocal cords and unremoved tongue base tissue does not affect the hyoid-larynx complex. However, the benefits of UPPP alone for OSA are unclear (12, 13). In view of the continuous updating of the literature on the increase in the prevalence of OSA (14, 15) and consumption of medical resources (16), requirements for surgical treatment of OSA have become more stringent. In comparison with previous surgical methods, TORS for OSA improves outcomes. From a review of the literature, use of a mechanical arm to treat the tongue base was first reported in 2006 (17). In 2012, Friedman compared three mainstream tongue base reduction surgical procedures (18): TORS for base of tongue (success rate 60.5%), submucosal wound tongue root excision surgery RFBOT (success rate 37%), and tongue root radiofrequency surgery (success rate 32%). Robotic surgery can increase the minimum blood oxygen concentration by 8%. In 2014, Vicini published a systematic review of 201 cases, which showed that TORS for OSA results in 53.8% of patients being removed from positive pressure respirator, with a cumulative success rate of 66.9%. D'Agostino et al. also agreed with the benefits of tongue base surgery and advocated the removal of the genioglossus muscle at a depth of 1 cm with a width of 15 cm on both sides and even to a depth of 1.5 cm within the central 2 cm (10). Lin and Hsu used intraoperative ultrasound to probe both sides of the lingual arteries or imaging studies for marking margins to determine the safe distance for tongue base surgery (19, 20). The above studies present the operation efficiency and improvements in operation safety, with no mention of the effects on voice or phonation quality after surgery.

Pronunciation is mainly regulated by endogenous laryngeal muscles. However, the external muscles of the tongue and larynx play an important role in ensuring the position of the hyoid bone-larynx complex and the internal structure of the larynx (5). Any hyoid bone shift is transmitted to the thyroid cartilage and tissues of the larynx. The inferior fiber of genioglossus muscle acts directly on the hyoid bone (5) with contraction of the posterior fiber of the genioglossus muscle causing the hyoid bone to move and the thyroid cartilage to slant forward, increasing the longitudinal tension of the vocal cords.

After removal of the dorsal area of genioglossus muscle, does minor adjustment to the position of the hyoid bone due to muscle weakening or iatrogenic surgical scar traction in stationary state and altered tension of the vocal cords persist? Are there any subjective or objective pronunciation problems post-surgery caused by remodeling of resonant cavity? These questions are related to post-operative quality of life.

The reason for using MDVP is that sound parameter analysis has widespread clinical applications such as in speech rehabilitation before and after surgery or radiation treatment and screening of larynx diseases. Bhuta explored the relationship between acoustic variables and perception and demonstrated that noise parameters may be perceived by patients with dysphonia, such as NHR and problems in VTI and SPI (6).

Yiu proposed that Jitter, Shimmer, and noise are related to subjective perception of sound (21). Parsa suggested that NHR better predicts postoperative sound quality and that a rise in NHR of more than 83% is indicative of a sound lesion (22). In this study, sound parameters were objective data. There were no significant differences in sound parameters before and after TORS for OSA.

Some studies have revealed contradictory results. Carding et al. did not advocate the use of computerized acoustic analysis system as a single test for identifying voice disorders (23). Albantia reported that the sensitivity of the Voice Disorder Index Scale, in comparison to the analytical results of acoustic parameters, is 29% and the specificity is 73%, with good internal consistency with voice performance questionnaire. Therefore, we added VHI-10 to provide subjective information on voice disturbance and dysphonia that is more accurate than the subjective judgment of audiologist (9) and to assess subjective satisfaction. William noted that a change in scale score of more than 4 points is meaningful (24). VHI-10 scores of patients in this study did not differ significantly before and after surgery.

Albantia demonstrated that sound parameter analysis can identify patients with or without sound issues. Some parameter changes, such as in frequency, amplitude, Jitter, and Shimmer, may not be perceived by patients (9). Wolfe et al. proposed that changes in objective sound parameters are not related to subjective voice quality (25). To further understand the factors that affect the subjective perception of patients, we analyzed the factors related to VHI-10 score increase. Most sound parameters were not related to VHI-10 score shift. This result is consistent with the findings of Albantia and Wolfe.

In terms of pitch change, the highest pitch increased from 353.18 ± 161.92 Hz to 387.99 ± 143.59 Hz. The lowest pitch increased from 117.45 ± 30.45Hz to 131.42 ± 48.44 Hz and the entire pitch range increased from 235.72 ± 156.70 Hz to 256.57 ± 104.30 Hz. Shift in pitch showed no statistical significance and no correlation with VHI-10. After converting the frequency to semitone (note), the highest note reached increased from 15.7 ± 8.90 to 18.7 ± 5.74 and the entire range of notes increased from 14.14 ± 8.23 notes to 16.14 ± 3.76 notes. The lowest note increased from 1.57 ± 1.51 before surgery to 2.57 ± 3.74 after surgery. Only the lowest note change before and after the operation showed a negative correlation with the change in VHI-10 score (Spearman's rho r = −0.808, *p* value = 0.028). That is, if the value of the lowest note decreased after surgery, it was related to an increase in the total VHI-10 score. Due to such conversion, there was a change from continuous variable (frequency) to sequence variable (note). Given that there are 3 octaves, the notes 1 to 36 are easily perceived by most people. For example, a reduction of 100 HZ, such as 988 Hz to 880 Hz is equivalent to a decrease of 3 semitones (B_5_, ${\text{A}}_{5}^{\#}$, A_5_) and 3 notes (36 to 34). However, at lower frequencies, a 100 Hz decrease, such as from 246 Hz to 146 Hz, is equivalent to up to 10 semitones (B_3_, ${\text{A}}_{3}^{\#}$, A_3_, ${\text{G}}_{3}^{\#}$, G_3_, ${\text{F}}_{3}^{\#}$, F_3_, E_3_, ${\text{D}}_{3}^{\#}$, D_3_) and 10 notes (12 to 3). Therefore, when frequency changes, the note changes at low frequency are larger than at high frequency, meaning that they are more easily perceived, as revealed by VHI-10 scores.

After the operation, the increases in highest and lowest frequencies were not statistically significant. It is likely that removing part of the genioglossus muscle causes the hyoid bone to move forward, increasing the tension of the vocal cords and, thus, increasing the frequency. We are far from understanding the relationship between muscle structure and function of the tongue (4). This may require more sample analysis or imaging support in the future.

## Limitations

This is a pioneering study. The sample is not large, which may have led to increased type I fallacy and reduced test force. At the same time, the multivariate regression model could not be used to explore the main influencing factors of the differences in voice disturbance index before and after surgery. Item-related patterns were used the mode of double variables related.

## Conclusion

In this single-institutional preliminary study, we demonstrated that TORS for OSAS is a feasible and safe treatment that preserves the voice. No significant changes in objective sound quality or sound range were found. There was also no correlation with VHI-10 score. Low frequency changes may be related to subjective satisfaction.

## Data Availability Statement

The raw data supporting the conclusions of this article will be made available by the authors, without undue reservation.

## Ethics Statement

The studies involving human participants were reviewed and approved by the IRB of the Human Experiment Committee of Chung Shan Medical University (CS-17104). The patients/participants provided their written informed consent to participate in this study.

## Author Contributions

H-HH conceived and designed the study. JW provided administrative support. C-HT analyzed and interpreted the data. C-HT and H-HH contributed by writing the manuscript. All authors were involved in collection and assembly of data and approved the final version of the manuscript to be published.

## Conflict of Interest

The authors declare that the research was conducted in the absence of any commercial or financial relationships that could be construed as a potential conflict of interest.
